# Anchored Design of Protein-Protein Interfaces

**DOI:** 10.1371/journal.pone.0020872

**Published:** 2011-06-17

**Authors:** Steven M. Lewis, Brian A. Kuhlman

**Affiliations:** Department of Biochemistry and Biophysics, University of North Carolina at Chapel Hill, Chapel Hill, North Carolina, United States of America; University of South Florida, United States of America

## Abstract

**Background:**

Few existing protein-protein interface design methods allow for extensive backbone rearrangements during the design process. There is also a dichotomy between redesign methods, which take advantage of the native interface, and *de novo* methods, which produce novel binders.

**Methodology:**

Here, we propose a new method for designing novel protein reagents that combines advantages of redesign and *de novo* methods and allows for extensive backbone motion. This method requires a bound structure of a target and one of its natural binding partners. A key interaction in this interface, the anchor, is computationally grafted out of the partner and into a surface loop on the design scaffold. The design scaffold's surface is then redesigned with backbone flexibility to create a new binding partner for the target. Careful choice of a scaffold will bring experimentally desirable characteristics into the new complex. The use of an anchor both expedites the design process and ensures that binding proceeds against a known location on the target. The use of surface loops on the scaffold allows for flexible-backbone redesign to properly search conformational space.

**Conclusions and Significance:**

This protocol was implemented within the Rosetta3 software suite. To demonstrate and evaluate this protocol, we have developed a benchmarking set of structures from the PDB with loop-mediated interfaces. This protocol can recover the correct loop-mediated interface in 15 out of 16 tested structures, using only a single residue as an anchor.

## Introduction

Because so many human diseases are caused by dysregulation of proteins or protein-protein interactions, the need to experimentally or therapeutically adjust these systems is great. A powerful tool for probing protein networks is other proteins engineered to bind particular naturally-occurring target proteins and modify or illuminate their behavior. To that end, many authors have introduced computational methods for creating these tool proteins, including both de novo binding partners and redesigns of existing interfaces.[1] One modeling suite used for this purpose, and many others, is Rosetta.[2]

Placing past successes in context, it remains quite challenging to create binding partners with desired functionality, and even minor successes are not routine.[3], [4] This is because interface design combines all the challenges of protein design, itself an incompletely solved problem, with the additional complication of docking orientation between the two proteins.

The protein design problem requires decisions on how to best search the sequence space, how (or even if) to best search the backbone conformational space, and how to combine the two searches efficiently.[5] In Rosetta, flexible design methods are often only iteratively flexible: their design protocol runs on a fixed backbone, and then backbone flexibility is modeled using a fixed sequence. This is because the algorithmic optimizations necessary to efficiently sample conformations and sequences preclude sampling both simultaneously. Recent flexible-backbone design methods modifying protein interfaces or loops include methods using local backbone minimization [6] and fragment insertion plus loop closure and design.[7], [8]

A second decision must be made when designing new interfaces: which proteins should interact? The two major methods for protein interface design include *de novo* design, which creates an interface between previously non-interacting proteins, and interface redesign, which modifies the properties of existing interactors or homologs thereof. *De novo* designs offer the opportunity to engineer new functions into the interaction, at the great cost of having to create the interface from scratch.[6], [9], [10] Redesigns offer the opposite tradeoff: there is an interaction in place to start from, but the designs are restricted to modifying existing functions by increasing affinity [11]–[15] or altering specificities [16]–[18].

Here we propose a new method offering a blend of these strengths which we call AnchoredDesign. The method has been implemented as a protocol in the Rosetta3 software suite.[19] The method creates an interface between an arbitrary (and arbitrarily functional) scaffold and a target, but it also creates the interface along a known interacting surface of the target, using information from a preexisting binding partner. The method accounts for backbone flexibility at the interface by iterating between loop remodeling and design.

This method requires a known structure of the target complexed to some binding partner, as well as a structure of the desired scaffold. The scaffold should have flexible surface loops amenable to design, and otherwise be chosen for desirable experimental characteristics. For example, the fibronectin domain type 3 repeat 10 (10FNIII or FN3) scaffold used by many researchers is an appropriate design scaffold.[20]–[25]

**Figure 1:**
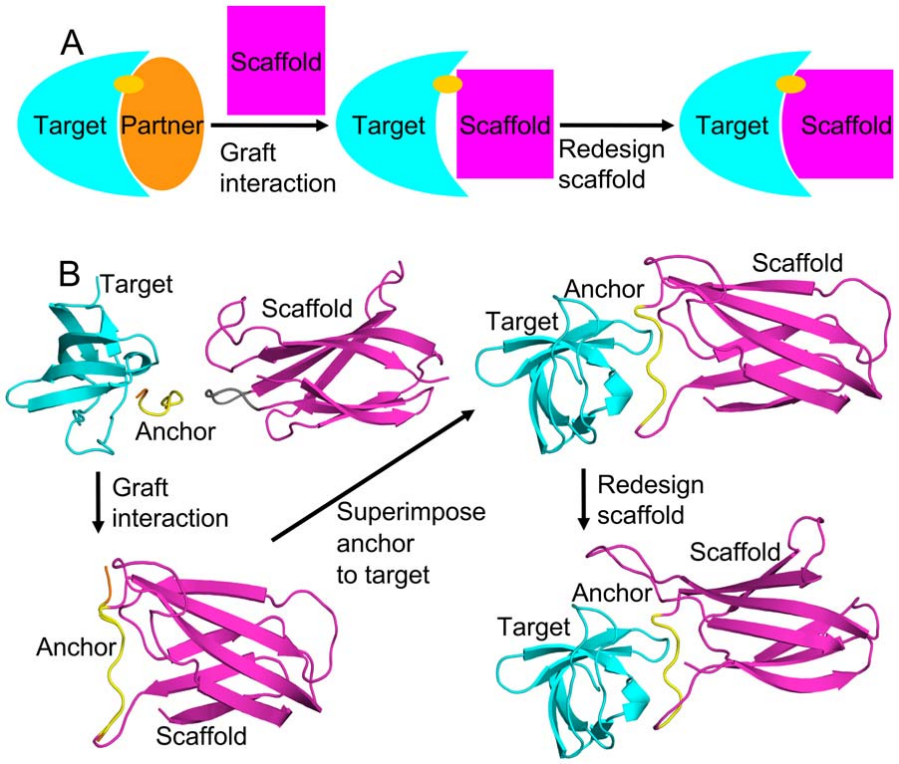
Anchor insertion. Panel A demonstrates the AnchoredDesign process with a simple cartoon. At left, we start with a known interaction between a target (cyan) and a natural partner (orange) with a characteristic interaction (the anchor, yellow). In the middle, we graft the anchor into the scaffold (magenta) to create a rough starting structure. At right, we fill out the scaffold-target interface with the AnchoredDesign protocol. Panel B demonstrates the process using protein structures for greater clarity (using the same color scheme).

The first step of this new method is to create a nascent interface between the target and the scaffold, as described in Figure 1. A small sequence-contiguous portion of the target's known partner is extracted, and its sequence identity and coordinates are inserted into a surface loop on the scaffold. This becomes the anchor. Standard Rosetta loop closure techniques can be used to close the scaffold's modified loop.[26] This results in an intermediate structure containing the target, the scaffold, and a small interface between them where the scaffold mimics the original binding partner. It is ripe for flexible redesign to create a real interface between the partners. Note that the use of this anchor guarantees that the new designed binder will bind to a surface area of the target overlapping the original partner's area. This helps control the activity of the new binder by ensuring that experimenters know where it is binding. It also controls for the residue composition of the target surface as suggested by Lo Conte et al.[27]

Grafting interactions into interfaces is not unknown in the literature, suggesting that the grafted anchor is likely to function as hypothesized. Potapov et al. searched for noncontiguous protein fragments (clusters of residues) from the Protein Data Bank (PDB) [28] matching the known backbone structure of an interface, and showed that mutating a new cluster into a pre-existing interface resulted in the maintenance of stability and specificity.[29] Liu et al. performed the opposite experiment: they created a new interface between shape-compatible but nonbinding proteins by grafting three residues from one interactor's normal partner onto the new binding partner.[30]

**Figure 2:**
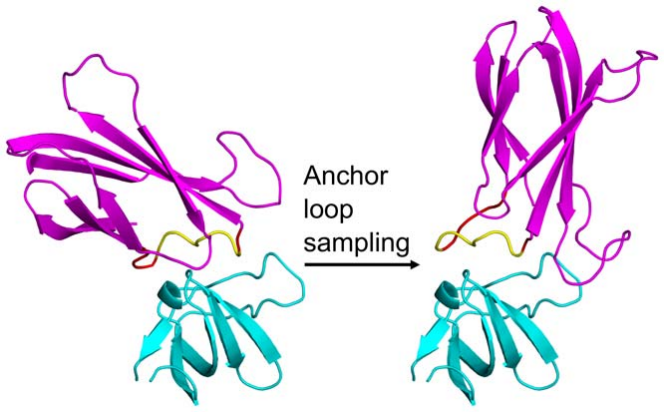
AnchoredDesign treatment of rigid-body and loop degrees of freedom. These complexes demonstrate how the AnchoredDesign protocol samples the rigid-body degree of freedom via loop sampling. These complexes are colored as in Figure 1: cyan for the target, yellow for the anchor, and magenta for the scaffold. The flexible residues of the loop containing the anchor are colored red. The two complexes are related only by the alteration of the backbone torsion angles of the red positions; the overall viewpoint has not been rotated (notice the targets are identical). Remodeling of this loop (and no other changes) produces a large rigid-body like change between the two partners, while leaving the anchor/target interface and both protein cores intact. This allows sampling of the target/scaffold interface without losing the anchor information.

After creating a nascent interface via grafting, the next step is flexible redesign. Normally one considers the docking problem when thinking about designing protein-protein interfaces. Here, the anchor precludes the use of whole-protein rigid-body motion as in docking, because this would cause the loss of the anchor. Instead, loop remodeling of the loop containing the anchor is used to sample the rigid body space between the proteins, as in Figure 2. Holding the anchor in its original binding conformation, rigidly affixed to the target, and remodeling its loop will result in rigid-body transformations between the target and scaffold. This allows us to use loop modeling to generate backbone flexibility at the interface and simultaneously sample possible binding modes of the scaffold. Other surface loops on the scaffold can be concurrently sampled to produce further surface complementarity.

For computational methods like these, benchmarking tests both help develop the protocol and demonstrate its utility. For the AnchoredDesign protocol, we have assembled a set of 16 protein structures from the PDB. These structures were chosen on the basis of having an interfacial loop with an appropriate residue to serve as an anchor. The protocol can then be tested against these structures by deleting the conformation of the anchor-containing loop and using the protocol to predict the proper binding orientation of the two proteins. This serves as a test of the loop modeling and interface predictions of the protocol. Here we present the protocol itself, as used for design or in these benchmarks, and the results of these fixed-sequence structure prediction benchmarks.

## Methods

### AnchoredDesign protocol

**Figure 3:**
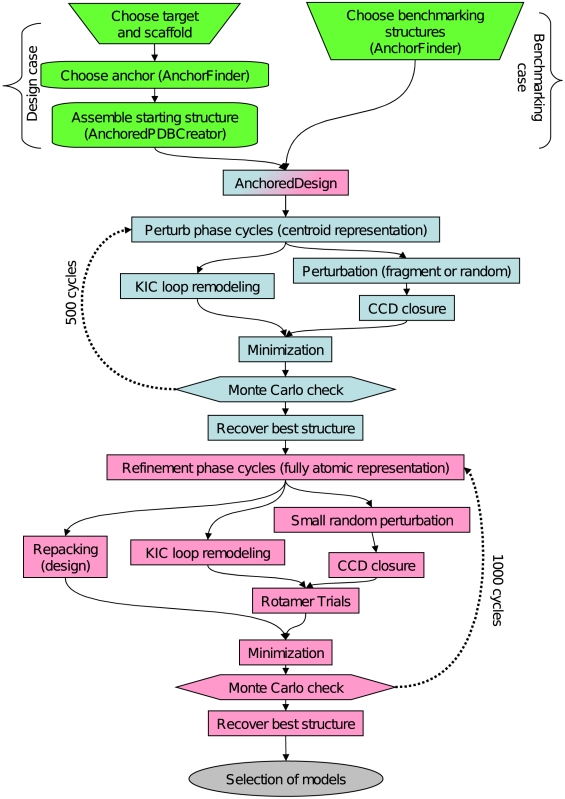
Protocol flowchart. This flowchart summarizes the AnchoredDesign protocol and process. In the top part, preliminary steps are marked in green. These steps are primarily manual but can be assisted with AnchorFinder and AnchoredPDBCreator. Initial steps vary depending on whether the benchmarking case for this paper, or the more general design case, is being addressed. The steps of the AnchoredDesign protocol are shown in blue and pink. The perturbation steps, performed in centroid mode, are in blue. The refinement steps, performed with a fully atomic representation, are in pink. In both portions of the protocol, many Monte Carlo cycles are performed; the results here used 500 perturb and 1000 refine cycles, but optimal cycle counts are best determined on a per-experiment basis.

The AnchoredDesign protocol is written as an application in the Rosetta3 software suite, and first released with the 3.3 release. It was designed from the ground up within the Rosetta3 framework and thus takes advantage of all the modularity and ease-of-use offered by that foundation.[19] The protocol uses a multistage Metropolis Monte Carlo search protocol, with large perturbational movements in a reduced centroid representation and smaller refining changes in a higher-resolution fully atomic phase. This sort of multistage centroid/fullatom protocol is common for Rosetta protocols.[26], [31], [32] Conceptually, the centroid phase is meant to sample conformational space widely and jump over relatively high energy barriers between conformations, whereas the fullatom phase is meant to minimize a centroid candidate structure into its local energy minimum. To accomplish this, the protocol iteratively samples loop conformations and sidechain conformations, with interspersed opportunities for design. Figure 3 offers a diagram of program flow summarizing the major steps of the protocol.

The first phase of the protocol is the centroid sampling phase, using Rosetta's reduced-sidechain centroid representation and scorefunction.[31] Centroid mode reduces the complexity of the side-chain packing problem while the search function tries to consider larger changes to the protein structure. The centroid sampling phase consists of many Monte Carlo cycles of loop remodeling and minimization. Two types of loop remodeling can be performed here: perturbation followed by cyclic coordinate descent closure (CCD) [26], [33], or “kinematic” (KIC) loop remodeling [34], [35]. Note that for either case, loop modeling proceeds slightly differently than previously published to account for the anchor; see details below. No sidechain optimization is necessary during centroid-mode perturbation, so after loop closure the algorithm proceeds directly into gradient minimization.[31] Backbone torsions at flexible loop positions are minimized to ensure good loop conformations and to perfect loop closure in the CCD case.

The second phase of the protocol is the refinement phase, which uses a fully atomic representation of the proteins. The use of a fullatom scorefunction, along with smaller-scale changes tested by Monte Carlo, allows this phase to refine the candidate structure produced in the perturbation phase. Here, CCD and KIC loop remodeling are also both available, although the CCD steps are softened to suggest smaller protein changes (described below). After each loop closure step, a quick fixed-sequence rotamer relaxation is performed [36], followed by a gradient minimization. The design portion of AnchoredDesign is incorporated during the fullatom phase by performing a sequence design and/or rotamer repacking on the interface region at user-defined intervals between loop remodeling cycles. Note that to reduce time spent repacking, all rotamer rearrangements used in AnchoredDesign feature automatic detection of the relevant residues: loop residues, their neighbors, and interface residues are automatically included, whereas residues outside those regions (the protein cores and distal surfaces) are not modified during repacking.

#### Loop modeling for AnchoredDesign

The KIC loop modeling protocol has been modified slightly from its original published implementation to allow for the constancy of the anchor. Normally, KIC solves an equation to determine phi and psi torsions for 3 loop residues, the pivots. The solutions to the equation are those torsions that close the loop. KIC also optionally selects new values for non-pivot torsions.[34] The modifications used here allow for the anchor positions to be excluded from the list of allowable pivots and modifiable non-pivot torsions; they do not otherwise affect the underlying algorithm at all.

CCD loop closure has also been slightly modified to account for the anchor. Normally, CCD closes a broken loop by iteratively altering phi and psi angles to attempt to bring the broken loop ends together.[33] Here, the anchor's torsions are held fixed during CCD; it has no effect on the algorithm other than introducing inflexible regions which act as a particularly long bond.

Rosetta loop sampling with CCD is normally paired with a perturbation step which breaks the loop and introduces diversity.[26] Here, multiple methods are offered for perturbation before CCD closure. In the perturbation phase, the simplest method offered is randomization of the phi/psi angles (within Ramachandran constraints) of several residues in the loop. Other options include several varieties of fragment-based [31] perturbation: pregenerated fragment sets, automatically generated sequence-specific fragment sets, or automatically generated sequence-nonspecific fragment sets are allowed. The former options are more useful for structure prediction; the latter for design (where the final sequence is not known during the perturbation phase, so fragments of varying sequence are appropriate). In the refinement phase, large loop rearrangements are not desired, so only randomization of phi/psi angles, within a few degrees and Ramachandran constraints, is offered as a method of generating variation before CCD. Fragment insertion is not performed during the fullatom refinement.

**Figure 4:**
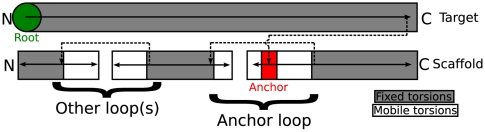
Fold tree diagram. This figure, modeled after the fold tree diagrams in Figure 1 of Wang et al. [26], demonstrates the kinematic connectivity that makes AnchoredDesign work. The arrows trace the direction of folding as Rosetta recalculates 3D coordinates from internal coordinates, starting at the green root residue. The upper and lower sections represent the target and scaffold respectively. Shaded regions represent rigid torsions (including the entire target and the core of the scaffold, in this case). Unshaded regions represent mobile torsions: the loops. All jumps between noncontiguous residues (dotted lines) are held rigid. AnchoredDesign embeds rigid torsions (the anchor, red) inside a loop, and affixes the anchor to the target by having the anchor's coordinates depend on the target instead of the scaffold in which the anchor is embedded. The scaffold is then dependent on the anchor via the mobile loop containing the anchor. Also allowed are arbitrarily placed other loops, handled with the standard loop fold tree.

Because AnchoredDesign is an interface design tool, but not quite a docking tool, it is necessary to explain how loop remodeling can effect rigid-body sampling without modifying the anchor or core of either protein. Figure 4 is a Rosetta fold tree diagram representing an AnchoredDesign fold tree, modeled after those in Wang et al.[26] Rosetta regularly updates atomic coordinates from internal coordinates (bond lengths, angles, and torsions) held by the atom tree and fold tree data structures.[19] The group of atoms moved by the rotation of any one bond is controlled by the connectivity of the atom tree, which is in turn set by the more general fold tree. In AnchoredDesign, the fold tree is built in such a way that the anchor residues are dependent only on the target protein, the anchor's loop depends on the anchor, and the scaffold (which is rigid around the loop) is dependent on the loop. This setup ensures that conformational changes to the anchor loop result in relative motion of the two proteins' cores: rigid-body sampling. Other surface loops are treated with a standard loop fold tree as in Wang et al.[26]

#### Methods for target selection

Because appropriate interfaces for testing the AnchoredDesign approach are only a small fraction of the available interfaces in the PDB, an automated method was created to find interfaces with loops resembling an anchored loop. This method has been released alongside AnchoredDesign as AnchorFinder within the 3.3 release. The AnchorFinder algorithm was written to help find appropriate benchmarking structures, but it can also suggest useful anchors against targets of biological interest.

AnchorFinder searches any number of input structures for the qualities that define an anchored interface. In particular, it searches for protein regions that are dominated by loop secondary structure (as determined by Rosetta's internal implementation of the DSSP secondary structure algorithm [37]) and contain large numbers of protein contacts involving two chains (which are therefore across an interface). AnchorFinder will output a listing of the DSSP assignment and cross-interface neighbors for each residue in each structure studied, plus summaries for contiguous regions that meet user-specified thresholds for length, secondary structure, and number of cross-interface neighbors. Regions with many cross-interface neighbors represent candidate anchors. When using AnchoredDesign to create new interfaces, AnchorFinder can help identify plausible anchors, but for small numbers candidate target/partner structures, manual examination is sufficient.

To choose our benchmarking set, the highest-ranking results from AnchorFinder were examined individually. AnchorFinder was run against the entire PDB (snapshot May 2009). The top several hundred structures returned by AnchorFinder were filtered to ensure that the hits were biological dimers and had identifiable anchors. The remaining hits contained redundant copies of many biological interactions due to multiple structures of some interactions, and multiple copies of one interaction within an asymmetric unit. Single representatives of each biological interaction were chosen. In general benchmarking systems were chosen to have a variety of biological sources, structures, and functions. One benchmarking structure, 2obg [24], was chosen for its identity as a fibronectin monobody structure without it appearing in the top fraction of AnchorFinder results: it represents the sort of structure AnchoredDesign is intended to create.

#### Choosing anchors, loops, and designable positions

AnchorFinder's results strongly suggest candidate anchors for use with AnchoredDesign. In general, the anchors used for benchmarking in this work were chosen by examining the loop residues suggested by AnchorFinder and picking one that either buried large amounts of surface area across the interface or choosing a residue with a cross-interface hydrogen bond. For the purposes of this benchmarking, only single-residue anchors were allowed, although the algorithm is compatible with longer contiguous anchors.

For the design case, anchors will be grafted into a different protein. It is therefore important to choose an anchor with some internal structure and/or a very well-defined interaction with the protein partner; examples might be 4 residues of a hairpin turn binding into a cleft or a phosphotyrosine binding an SH2 domain, respectively. Another possibility is the use of hot-spot residues [38], [39], including those determined by fast computational tools [40], [41]. Ultimately, anchor choice is a dimension of conformational space that must be searched by testing different anchors. Anchors can be evaluated computationally by examining the scores assigned by Rosetta to models using different anchors.

For the benchmarking presented here, the length for the remodeled loop containing the anchor was chosen by simply accumulating residues out from the anchor in both directions until non-loop secondary structure was encountered. Only this single loop was varied, although the code is compatible with multiple (non-anchored) surface loops on both sides of the interface.

In the design case, choice of flexible loops will be dependent on knowledge of the scaffold. Loops must have an absolute minimum of three mobile positions for KIC modeling to work.[34] Which loops and residues should be considered flexible, which scaffold loop should accept the anchor insert and at what position, and what length the loops should be must be determined manually by feeding different inputs to AnchoredDesign and comparing the quality of the resulting models.

Similarly, the choice of designable positions is dependent on knowledge of the scaffold. Scaffolds are presumably chosen on the basis of experimental experience with their tolerance to mutation (for example, fibronectin monobodies [22] or diverse other scaffolds [25]). The protocol assumes, but does not require, that the designable positions are all on flexible loops on one side of the interface (one-sided design). It will nevertheless accept two-sided design problems or non-loop design positions. Designable positions that are near neither flexible loops nor the interface may fail to be designed as desired, because the protocol automatically freezes those portions of the protein.

#### Creating starting structures

For the benchmarking in this paper, inputs for AnchoredDesign were generated from the crystal structure interaction with little modification. Nonprotein atoms (waters, cryoprotectants, and in some cases ligands) were deleted. These were passed through a simple structure minimizer to relax out any clashes with the Rosetta scorefunction. This protocol, InterfaceStructMaker (Peter Benjamin Stranges, unpublished protocol) performs a full-protein minimization and packing. It was determined that this preparatory step had no effect on the RMSD of the best scoring models (data not shown); its purpose was to remove data artifacts due to clashes in the crystal structures. These minimized structures were then fed directly to AnchoredDesign. AnchoredDesign internally deletes unwanted starting structure information (loop conformation, sidechains) when performing the benchmarks described in this paper.

In the design case, preparation of AnchoredDesign starting structures is much more complicated, because the anchor must be grafted from one structure into another. AnchoredDesign has a companion protocol also released with Rosetta3.3, AnchoredPDBCreator, designed to take care of this process. Two structures embodying three protein regions are necessary: a structure of the target protein with the protein containing the anchor bound, and a structure of the scaffold. Coordinates for the anchor, target, and scaffold are extracted into separate PDB files and offered as inputs to AnchoredPDBCreator, along with a file specifying what scaffold positions form the anchor loop and which positions the anchor should occupy. AnchoredPDBCreator inserts the anchor into the scaffold loop, closes the scaffold loop using CCD, and aligns the anchor (still rigid within the scaffold) with its binding site on the target. This resulting structure has the anchor and target correctly oriented (although the scaffold might interact poorly or eclipse the target), and is suitable as input to AnchoredDesign. The process is described in the first three subpanels of Figure 1, panel B.

#### Performing modeling

Workflow for AnchoredDesign is much like other Rosetta protocols: create and tweak input files, feed them to a cluster supercomputer to run tens of thousands of trajectories, then sift through the results. AnchoredDesign requires starting structures (outlined above), anchor and loop specifications (also outlined above), optionally a fragments file, and a resfile when performing design. These file formats and AnchoredDesign command line options are described in the Rosetta3.3 documentation. Briefly, options can be used to tweak the intensity of packing, control scorefunction and minimization settings, and control the length and temperature of the two Monte Carlo sampling phases.

For the benchmarking case, sufficient results to generate a score vs. RMSD metric plot are all that is required; this tends to be several thousand structures.

For the design case, the search space is much larger and the correct answer is not known, so generating many tens of thousands of structures for a particular design problem is appropriate. The protocol cannot perform insertions or deletions, or slide the anchor's position within the loops, so testing scaffold variants in this vein is highly recommended. It is also a good idea to use the results of one round of modeling to inform the next: if one round of modeling shows that a particular loop length never results in a tight interface, throw that series of structures out.

The starting structure produced by AnchoredPDBCreator is very rough and does not consider scaffold-target interactions. It is always necessary to run AnchoredDesign on these structures with sufficient perturbation-phase cycles to get a reasonable alignment of the two partners. Later modeling beginning from better structures can run through only the refinement phase (option AnchoredDesign::refine_only) to find the lowest energy sequences possible.

The optimum settings for the length of the perturbation and refinement phases of AnchoredDesign are system-specific. A good starting point would be 500–1000 perturbation cycles, followed by twice that many refinement cycles. The option AnchoredDesign::refine_repack_cycles controls how often a full repacking/design step is performed during the refinement phase; this option should not be less than 50 (more than that is designing needlessly frequently) and should not be more than 1/4 of the total refine cycles (or design is too infrequent).

#### Analyzing results

AnchoredDesign results are analyzed similarly to other Rosetta protocols'. The resulting structures and scorefile will contain the summed and individual scores, per-residue, for each term in the Rosetta scorefunction. Choosing the most likely models means choosing the lowest-scoring structures. AnchoredDesign also features a series of extra analysis tools to help highlight the better structures. These tools are implemented as Movers [19] which allows their analysis to be easily added to other protocols. Table 1 annotates the scorefile, and Figure S1 annotates the extra analysis output appended to result PDB files.

**Table 1:** Annotated scorefile headers.

Metric	Purpose
CA_sup_RMSD	Whole complex Cα RMSD after superimposition
I_sup_bb_RMSD	Interface main chain atom RMSD, after superimposition
ch1_CA_RMSD	Chain 1 Cα RMSD without superimposition
ch1_CA_sup_RMSD	Chain 1 Cα RMSD with superimposition
ch2_CA_RMSD	Chain 2 Cα RMSD without superimposition
ch2_CA_sup_RMSD	Chain 2 Cα RMSD with superimposition
loop_CA_sup_RMSD	Loop residues' Cα RMSD with superimposition
dSASA_int	SASA buried by the interface
dG_cross	Interface binding energy, calculated from residue interactions between chains
dG_cross/dSASAx100	dG_cross, scaled by dSASA_int and a constant factor
dG_separated	Interface binding energy, calculated by separating components
dG_separated/dSASAx100	dG_separated, scaled by dSASA_int and a constant factor
delta_unsatHbonds	Number of unsatisfied hydrogen bonds in the interface
total_score	Weighted, summed score of the scorefunction
LAM_total	A sensitive descriptor of loop closure quality
description	The trajectory label (e.g., 2OBG_0001)

This table annotates the regions of the scorefile produced by AnchoredDesign. The first column lists metrics useful for analyzing benchmark or designed structures, and the second lists the meanings of those metrics. Of particular interest are total_score, loop_CA_sup_RMSD (loop RMSD), I_sup_bb_RMSD (IRMSD), and ch2_CA_RMSD (LRMSD), which provide the metrics used for the other plots and tables in this paper. Scorefile columns not listed here are either scorefunction terms [31], [36] or InterfaceAnalyzerMover metrics not useful for AnchoredDesign.

InterfaceAnalyzerMover examines the quality of the interface in the final model. Included considerations are the burial of solvent-accessible surface area (SASA), the energy of binding, and the number and location of unsatisfied hydrogen bonds in the interface. These are important because AnchoredDesign optimizes stability of the complex (total energy), not binding energy.

LoopAnalyzerMover examines the quality of the flexible loops. It emphasizes the scorefunction terms relevant to loop closure (standard terms rama, dunbrack, and omega, [31] along with the chainbreak [26] term. It also prints the torsion angles of loop residues and the peptide bond distances. These data make it easy to spot poorly closed loops underpenalized by the Score12 scorefunction. These data are included at the end of the PDB output, as shown in Figure S1.

The benchmarking presented here also triggers an extra suite of RMSD analyses which examine the similarity of the result structure to the correct complex. These include the RMSD fields in Table 1, and are further discussed in the Results.

## Results

### Selected models

In order to test the AnchoredDesign protocol, we used the AnchorFinder protocol to search for protein dimers with naturally-occurring anchor sequences where a residue of one partner is deeply buried into the other partner and part of an interfacial loop. Table 2 lists the structures' identities along with the anchors and loops chosen for benchmarking. All anchors are single residues. Loop length varies from 8 to 16 residues. Represented structures include homodimers of various functions (1fc4, 1qni, 2qpv, 3dxv, 1u6e, 2bwn, 2hp2, 2wya, 3cgc, 3ean, 1fec), two antibody/antigen complexes (1jtp, 2i25), one enzyme/inhibitor complex (1zr0), one engineered binder/target complex (2obg), and one nonbiological crystal dimer (1dle) chosen as a test of weak interactions. The crystal structures' resolution ranges from 1.7–2.75 Å, and the SASA buried in the interface ranges from 1400 to 11,800 Å^2^.

**Table 2:** Input structures and accessory data.

PDB	Chains	Res. (Å)	Dimer type	SASA (Å^2^)	Anchor	Chain	Loop	Loop length
1dle	A/B	2.1	crystal dimer**	2,800	38	B	36–40***	8
1fc4	A/B	2	homodimer	10,000	74	B	69–79	11
1qni	A/B	2.4	homodimer	11,800	408	B	397–411	15
2qpv	A/B	2.35	homodimer	2,900	55	B	50–57	8
3dxv	A/B	2.21	homodimer	7,900	291	B	288–299	12
1u6e	A/B	1.85	homodimer	6,700	86	B	80–89	10
2bwn	A/B	2.1	homodimer	8,500	85	B	77–90	14
1jtp	M/B	1.9	heterodimer	1,600	104	B	99–108	10
2hp2	A/B	2.7	homodimer	9,500	2306	B	2294–2309	16
2wya	B/C	1.7	homodimer	5,900	1009	C	1003–1012	10
2obg	A*	2.35	heterodimer	1,600	1080	A	1077–1086	10
2i25	M/O	1.8	heterodimer	1,400	91	O	86–93	8
3cgc	A/B	2.3	homodimer	5,400	429	B	421–434	14
3ean	A/B	2.75	homodimer	7,900	473	B	467–474	8
1fec	A/B	1.7	homodimer	6,400	459	B	456–463	8
1zr0	A/B	1.8	heterodimer	1,400	15	B	10–17	8

This table collects structural parameters for the complexes used in this work, along with chosen parameters like anchor placement. The PDB column identifies the structure. The chains column identifies which complex within the PDB file was used (several have many complexes in the asymmetric unit). *: 2obg was not crystallized as a heterodimer; it was expressed as a fusion protein and crystallized as an infinitely domain-swapped polymer.[24] The resolution column contains the reported crystal structure resolution; all are reasonable. The dimer type column identifies the type of dimer. **: 1dle represents a crystal dimer rather than a biological one, making it a good test of weak interactions. The SASA column notes the area buried by the interface. The anchor and chain columns together identify the residue used as an anchor. The loop column identifies which residues (on the same chain as the anchor) were flexible. The loop length column collects the lengths of these loops. ***: 1dle has residues with insertion codes in the loops, leading to a longer loop than is obvious. The name column identifies the name and function of the protein as listed in the PDB.

### Overall quality of predictions

The AnchoredDesign protocol was challenged with a benchmark where it was given a dimer structure with the rigid-body orientation, interface side chains, and an interfacial loop's conformation deleted. With knowledge of the position and conformation within the interface of one residue (the anchor) of the deleted loop, AnchoredDesign was asked to predict the correct loop structure and rigid-body orientation of the two proteins. Due to the fixation of the anchor, the backbone degrees of freedom and rigid-body orientation are treated simultaneously by loop closure (Figures 2 and 4). Beyond this prediction experiment, two further experiments were performed to diagnose the source of failed predictions and provide performance comparisons. In one, the starting structure's loop and side chain information is not deleted: the simulation starts at the correct answer; the test is whether and how far the result drifts from the correct starting structure. These are often called “relaxed natives”. In the second, the same information is not deleted, plus the AnchoredDesign protocol is instructed to skip the broad-sampling centroid perturbation step, and perform only the high-resolution refinement step. This is a more conservative calculation of the relaxed native population. If differences between these two forms of relaxed natives occur, it indicates that data are being lost during the centroid phase due to the low resolution of that protein representation.

AnchoredDesign's prediction of the interface was measured with three root-mean-square deviation (RMSD) metrics. The first metric was Cα RMSD of loop residues after superimposition, which gives a measure of how well the crystal loop was recapitulated. The second was backbone atom RMSD (after superimposition) of residues found at the interface, IRMSD, which measures how well the shape of the interface was recovered. The third metric was Cα RMSD for all residues on the moving side of the interface, LRMSD. This was calculated with superimposition on the nonmoving side of the interface, and thus gives a measure of whether the moving side of the interface is placed in its proper rigid-body position and orientation by AnchoredDesign. IRMSD and LRMSD are approximately equivalent to the metrics used in the communitywide CAPRI docking prediction experiment for gauging the quality of docked interfaces.[42] These three RMSD calculations are labeled loop_CA_sup_RMSD, I_sup_bb_RMSD, and ch2_CA_RMSD in AnchoredDesign output (Table 1, Figure S1).

**Figure 5:**
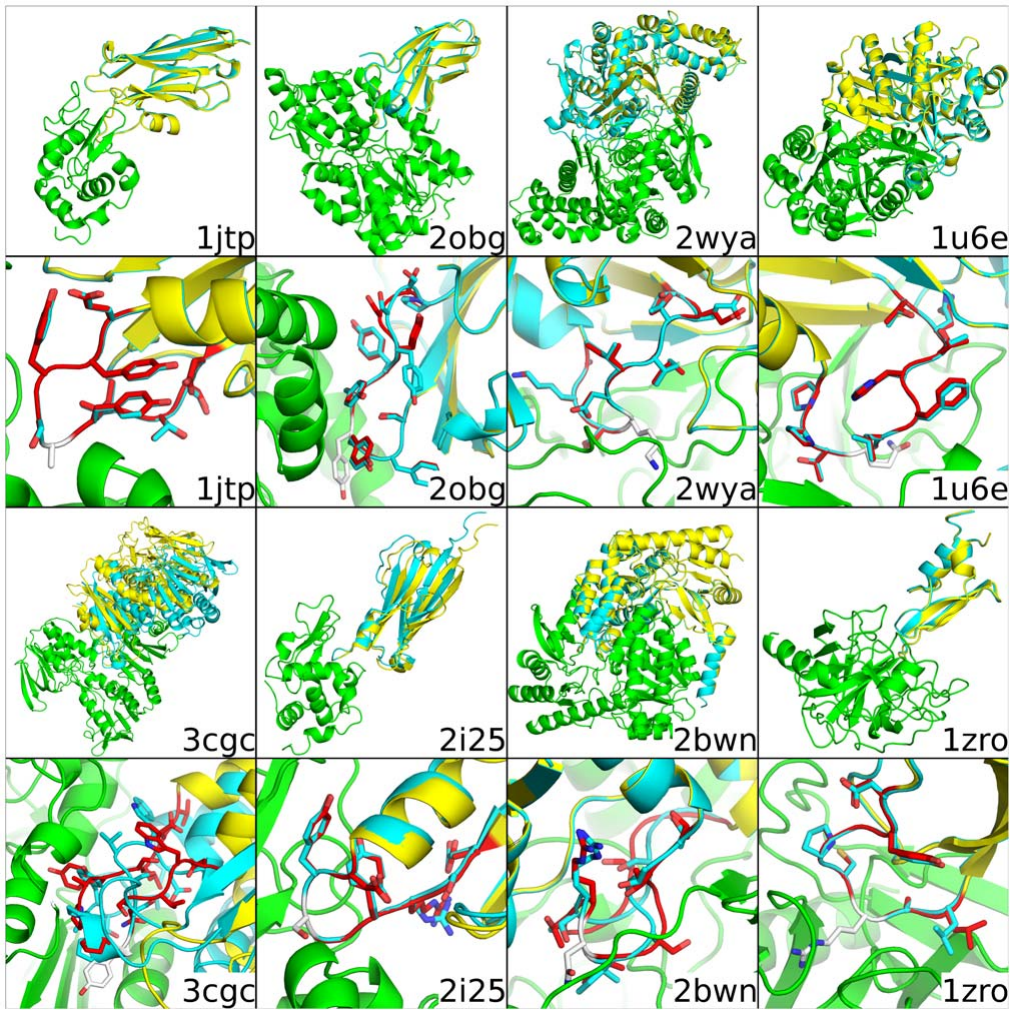
Best scoring prediction for 8 complexes. This figure demonstrates the relaxed crystal structure input and AnchoredDesign's lowest-score prediction for 8 of the 16 structures. The first and third rows show whole structures, and the second and fourth zoom in on the predicted loops. The nonmoving side of the interface is in green, the actual partner in cyan and the prediction in yellow. The predicted loop is red and the anchor is white. Structures are labeled with their PDB code in the lower right of each cell. For most structures, the predicted rigid-body placement and loop is indistinguishable from the relaxed crystal structure; 3cgc (lower left) is the exception. The other 8 structures are shown similarly in Figure S2.

Table 3 lists the RMSD metrics for the lowest-score structure predicted by AnchoredDesign for each input (compared to the minimized crystal structure used as input). In most cases, AnchoredDesign produces extremely accurate models for which each metric is below 1 Å RMSD; exceptions are further discussed below. Figures 5 and S2 show each of the 16 complexes, including the minimized crystal structure and lowest-scoring result from AnchoredDesign. For most cases, the prediction is indistinguishable from the correct structure. Note that lowest-scoring is defined purely by Rosetta's standard Score12 scorefunction, plus a chainbreak term used in CCD loop modeling; these weights are listed in Table S1.

**Table 3:** RMSD of lowest-scoring models.

PDB	Input loop RMSD (Å)	Crystal loop RMSD (Å)	Input IRMSD (Å)	Crystal IRMSD (Å)	Input LRMSD (Å)	Crystal LRMSD (Å)
1dle	0.09	0.42	0.04	2.24	0.16	1.69
1fc4	0.40	0.43	0.08	0.57	0.08	0.70
1qni	1.99	1.98	0.53	0.94	0.47	1.00
2qpv	0.63	0.68	0.19	0.78	0.23	0.97
3dxv	0.62	0.65	0.14	0.55	0.13	0.60
1u6e	0.13	0.35	0.06	0.59	0.12	0.62
2bwn	1.30	1.35	0.30	0.75	0.29	0.86
1jtp	0.08	0.29	0.07	0.73	0.30	0.97
2hp2	3.43	3.43	1.37	1.52	1.24	1.45
2wya	0.24	0.28	0.06	0.54	0.06	0.88
2obg	0.34	0.36	0.26	0.61	0.58	0.74
2i25	0.11	0.28	0.28	0.61	2.18	2.28
3cgc	2.23	2.26	4.74	4.90	14.16	14.41
3ean	0.06	0.38	0.03	0.91	0.06	0.91
1fec	0.14	0.39	0.03	0.60	0.04	0.72
1zr0	0.17	0.40	0.14	0.47	0.47	1.63

This table summarizes predictions of the AnchoredDesign benchmark. Each value represents the RMSD of the lowest-scoring model produced by AnchoredDesign for that structure using its standard protocol. The PDB column identifies the structure. The loop RMSD, IRMSD, and LRMSD columns describe the RMSD of the lowest-scoring prediction against the relaxed input structure. The input columns compare AnchoredDesign's output to the scorefunction-minimized crystal structures used as input. The crystal columns compare the same output to the unrelaxed crystal structures. The low values throughout indicate that AnchoredDesign does a good job recovering native interfaces starting from extended loops. The similarity of the input and crystal columns indicates that the relaxed starting structures are not far from the crystal structures.

**Figure 6:**
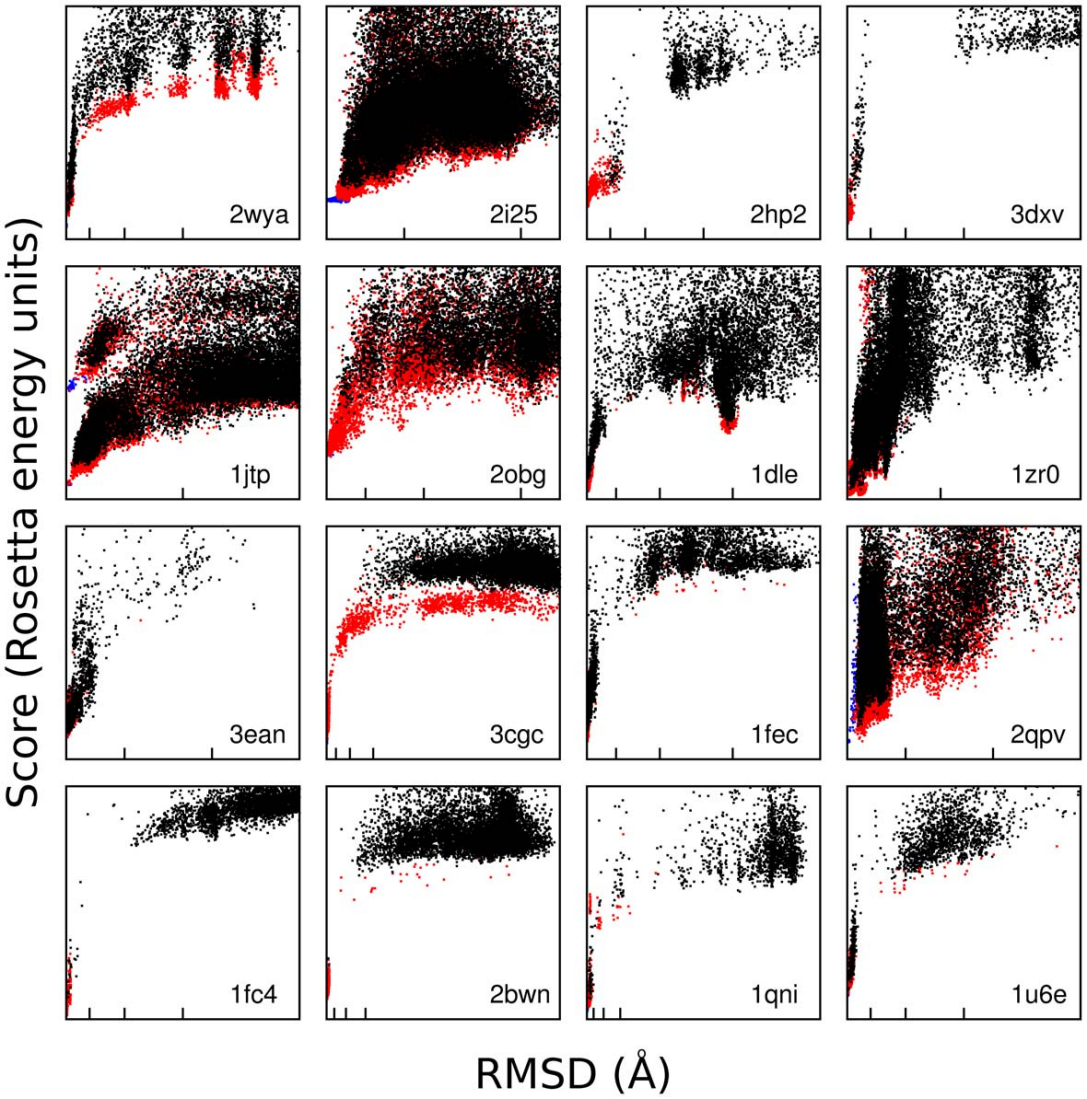
IRMSD versus score plots. This figure shows score versus IRMSD plots for each of the 16 structures. RMSD was calculated between the relaxed crystal structure (input) and AnchoredDesign's output. Plots are labeled with their PDB ID in the lower right of each cell. Black points are predictions, red points are relaxed native trajectories, and blue points are conservative relaxed native trajectories which skipped the centroid phase (see main text). Blue points may lie under red points, and red points may lie under black points. Some high-score points are out of view on all plots; all low-score points are present. On the RMSD (X) axis, the first, second, and third tic marks represent 1, 2.5, and 5 Ångstroms. Some plots are zoomed in beyond 5 or 2.5 Ångstroms and fewer tics appear.

Figures 6, S3 and S4 show score versus RMSD plots for each structure for each of the three metrics. These plots demonstrate that AnchoredDesign produces “funnels” for most of these experiments: all low-energy points are also low-RMSD, and RMSD rises with energy. This indicates the scorefunction grades these structures accurately and AnchoredDesign samples possible structures effectively. To visualize the space which AnchoredDesign samples, Figure S5 shows the result of 100 trajectories for two PDBs (2obg and 1fc4). The protocol clearly samples many possible interfaces and rigid body orientations, but is nevertheless able to determine which is correct.

For most experiments, these predictions required 1 day on 128 2.33 GHz processors per experiment, which produces thousands to tens of thousands of trajectories depending on the size of the input structure. Some experiments required extra computer time to accommodate larger proteins. Similar quantities of sampling were used for the relaxed native experiments; only 512 models per structure were produced for the conservative, fullatom-only relaxed natives.

### Sampling errors

The most significant failure in this benchmark is the inability of AnchoredDesign to predict a correct interface for structure 3cgc. This structure is of a bacterial Coenzyme A disulfide reductase.[43] Figures 6, S3 and S4, panel 3cgc, show no low-RMSD points and no real score discrimination for the prediction experiment (black points). When the loop is not deleted prior to prediction, lower score, low-RMSD conformations are created (red and blue points). This demonstrates that it is not a flaw in the fullatom scorefunction but rather in sampling: the protocol never examines a loop resembling the correct loop, but it does give low scores to correct loops for relaxed natives. The relaxation experiment (red points), which runs AnchoredDesign as normal on an intact input loop, can be seen to hop out of the score well for correct structures and produces a smear of isoenergetic high-RMSD points. The fact that relaxed natives can lose their correct conformation implies that the problem may be a combination of errors. It could be that the low-resolution centroid scorefunction is unable to recognize the correct structure, and the fullatom phase's sampling is insufficient or ineffective in recovering low-RMSD structures for 3cgc.

### Loop conformation errors

**Figure 7:**
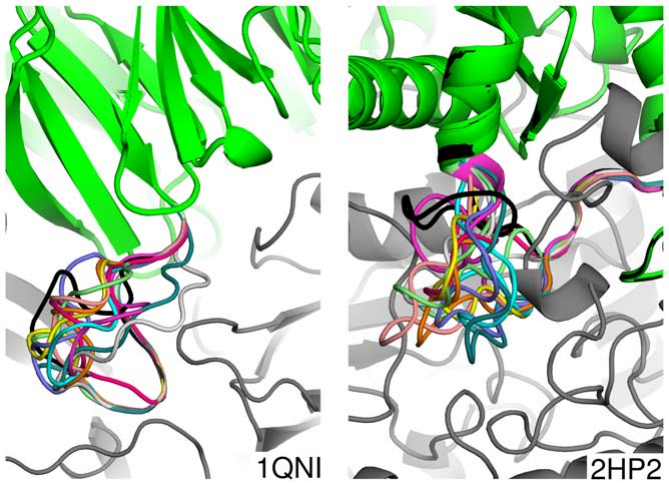
Poorly predicted 1qni and 2hp2 loops. This figure shows the insufficiency of AnchoredDesign's loop predictions for 1qni and 2hp2. In gray is the nonmoving side of the interface and in black is the correct structure. Each of the ten colors represents the loops of one of the top ten predictions by lowest energy. The predictions' protein cores are green. Notice that the loops themselves do not converge, but that the rigid-body placement and interface as a whole is correct (the green and black portions are overlaid towards the top of each panel).

Structures 1qni and 2hp2 represent a pair of partial failures. These two structures score well on IRMSD and LRMSD metrics, but have relatively poor predicted loop RMSDs. In these two cases, AnchoredDesign finds and recognizes the correct interface between proteins without folding the anchor loop correctly. Figure 7 shows the ten lowest-energy predictions for these two structures. In each case, all structures have the correct interface, but the loop itself is not predicted correctly, and does not converge onto a single prediction. Apparently, the energy well containing the correctly-bound interface is deep enough that the scorefunction can find it through minimization of loop degrees of freedom without accurately sampling the loop itself. Figure S4 indicates that AnchoredDesign is probably failing to sample the correct loop in both cases, because the conservative relaxed natives (which maintain the correct loop conformation) are lowest RMSD and lowest scoring. This is probably related to the fact that these loops are longer than most loops in this benchmark (see Table 2, Discussion). AnchoredDesign's assignment of varied incorrect loops as isoenergetic is probably due to the lack of nearby steric restraints. The 1qni loop is mostly solvent-exposed, meaning that many solutions are possible. The 2hp2 loop borders a ligand absent during the modeling, freeing volume which then allows for many solutions.

### Rigid body placement errors

Table 3 shows that the three metrics are slightly inconsistent for structure 2i25, a shark antibody bound to lysozyme.[44] Specifically, the loop RMSD and IRMSD metrics indicate a correct solution, while the LRMSD metric indicates a deviation. Examination of this structure (Figure 5, panel 2i25) shows that the interface and the loop are nearly the same set of residues: the CDR3 loop of the antibody. The relatively elongated antibody fold and the presence of a C-terminal tail pointing away from the interface amplify tiny errors in loop structure between the interface and antibody core to produce a relatively large displacement of the opposite side of the antibody. The prediction shows clearly that AnchoredDesign is correct despite the slightly high LRMSD.

### Comparison of loop closure methods

To test whether CCD or KIC loop closure was more appropriate for AnchoredDesign, all experiments were repeated using only CCD or KIC loop remodeling. Three general trends were found. First, AnchoredDesign with only CCD sampling is usually slower than AnchoredDesign with only KIC sampling; the default protocol using both falls in the middle (data not shown). Second, for a few structures (2obg and 2i25), more trajectories were required with KIC sampling to get results equivalent to CCD or combined sampling. Finally, all three methods produce results of equivalent qualities, as shown in Table 4. We also found that structure 2bwn, which can be seen in Figures 6, S3 and S4 to rarely sample the correct conformation, samples the correct conformation even less efficiently with only one style of loop remodeling. Taken together, these results imply that the default protocol, using both methods, is most appropriate for the design case where the correct structure is not known. KIC-only closure offers a speed benefit but may not work as well on all structures.

**Table 4:** Comparison of loop closure methods.

	RMSD (Å) for default protocol			RMSD (Å) for CCD protocol			RMSD (Å) for KIC protocol		
PDB	loop	IRMSD	LRMSD	loop	IRMSD	LRMSD	loop	IRMSD	LRMSD
1dle	0.09	0.04	0.16	0.25	0.10	0.17	0.11	0.10	0.39
1fc4	0.40	0.08	0.08	0.34	0.09	0.12	0.50	0.11	0.10
1qni	1.99	0.53	0.47	2.85	0.60	0.56	2.21	0.48	0.43
2qpv	0.63	0.19	0.23	0.66	0.20	0.30	0.34	0.11	0.24
3dxv	0.62	0.14	0.13	0.51	0.13	0.12	0.78	0.20	0.18
1u6e	0.13	0.06	0.12	0.17	0.05	0.09	0.09	0.05	0.09
2bwn	1.30	0.30	0.29	1.77	0.40	0.58	1.80	0.36	0.39
1jtp	0.08	0.07	0.30	0.20	0.11	0.21	0.19	0.12	0.42
2hp2	3.43	1.37	1.24	4.56	1.60	1.44	4.68	1.43	1.28
2wya	0.24	0.06	0.06	0.71	0.14	0.12	0.12	0.02	0.03
2obg	0.34	0.26	0.58	0.45	0.33	1.05	0.47	0.36	0.70
2i25	0.11	0.28	2.18	0.22	0.40	3.17	0.11	0.24	2.00
3cgc	2.23	4.74	14.16	3.08	19.38	56.97	2.66	6.63	21.68
3ean	0.06	0.03	0.06	0.06	0.02	0.04	0.07	0.03	0.06
1fec	0.14	0.03	0.04	0.14	0.03	0.02	0.11	0.02	0.03
1zr0	0.17	0.14	0.47	0.10	0.07	0.70	0.06	0.04	0.20

This table demonstrates the rough equality of results from KIC, CCD, and combined loop sampling. The default protocol columns are the same as in Table RMSD; the CCD and KIC columns were generated using either loop modeling type alone. Each column represents one of the RMSD metrics described in the Results. Each of the loop modeling choices is broadly equivalent. Notice that structure 3cgc has in lower RMSDs under the combined protocol than either loop sampling type alone; this is a falsely optimistic interpretation because AnchoredDesign is never correctly predicting 3cgc under any of these protocols. The structures produced for 3cgc are isoenergetic and it is coincidental that the combined protocol happens to have lower RMSDs.

### Effects of anchor displacement

To test how sensitive our results were to the exact position of the anchor, we performed an experiment where the anchor was randomly displaced from its correct position. The protocol was modified to allow the anchor to move freely, to allow relaxation of clashes introduced by the random displacement. The anchor was gently constrained to its original position to prevent these initial clashes causing a total ejection of the anchor. Table S2 shows that AnchoredDesign is able to correctly predict the interface in most cases in this modified experiment. This demonstrates that AnchoredDesign is not hypersensitive to the exact starting conformation at the interface; small errors and flexibility in anchor placement do not pose a problem.

### Summary of results

Overall, these results are very encouraging for AnchoredDesign. Most structures tested are predicted with a very high level of accuracy, as seen in Figure 6 and Table 3. Note that none of the IRMSD versus score plots in Figure 6 demonstrate false funnels (there are no populations of low-energy, high-RMSD points). This indicates a lack of scoring failures, where incorrect structures are scored better than correct structures. Figure 6 also indicates that sampling failures are rare: only one case (3cgc) has no sub 2.5 Å RMSD points, and most cases have many sub-1 Å interface RMSD predictions. The few failures of the protocol can be attributed with some confidence to issues in the input (missing ligands, loop placement and length) rather than problems with the protocol itself. Additionally, the protocol is robust against small errors in anchor placement.

## Discussion

The novel AnchoredDesign protocol described in this paper is capable of predicting the proper conformation of loop-mediated interfaces, as demonstrated by its benchmarking performance against 15 of 16 structures. In these predictions, AnchoredDesign is able to assemble one fixed, correct backbone with another mostly fixed, mostly correct backbone by knowing one point of contact and performing loop modeling to search rigid-body and loop conformational space. AnchoredDesign's success should not come as a surprise: Rosetta and many other docking protocols have been proven to perform very well in fixed-backbone docking from bound backbones.[32], [42], [45] Rosetta has also succeeded at docking with loop remodeling.[26] That protocol was similar in its degrees of freedom to the one presented here, but different in its treatment of those freedoms. Recently, Rosetta-based docking algorithms were shown to correctly predict a trypsin/inhibitor complex like 1zr0 [46]; CAPRI Target 40, which Rosetta predicted at the highest level of accuracy.[47] We recently used AnchoredDesign to model an unknown interface between a fibronectin monobody and SH3 domain target, using a canonical polyproline binding interaction as an anchor.[48] We found that AnchoredDesign's models of fluorophore-tagged protein produced results consistent with experimental fluorescence.

The total (3cgc) and partial (1qni, 2hp2) failures of AnchoredDesign in this benchmark all share a common thread: these tests have loops longer than other, better-predicted complexes. These three tests have the longest loops at 14, 15, and 16 residues, respectively. Structure 2bwn, with a 14-residue loop, represents a borderline success. The lowest-energy structures are low in RMSD, but they are quite rare: careful examination of Figures 6, S3 and S4 (panel 2bwn) reveals only two low-energy points (both also low-RMSD) for structure 2bwn. For the other 12 structures that are clearer successes, the loops are 8, 10, or 12 residues. Loops were chosen not based on length but rather local structure characteristics: the loop length is the length of the loop in the native structure, chosen by extending from the anchor out to the closest sheet or helix. This dependence of result quality on loop length is not surprising; other authors have found similar dependencies with Rosetta's loop modeling protocols. The KIC protocol was proven to work well on loops of length less than 13 residues [34] and Rosetta's other loop prediction techniques also work better on loops shorter than 13.[49]

Benchmarking successes and failures aside, the purpose of AnchoredDesign is not to provide another docking tool to work on known interfaces; it is to provide an interface design tool for the creation of new interfaces. This work demonstrates the ability of AnchoredDesign to address the flexible-backbone interface prediction aspect of the interface design problem. A necessary ingredient not tested here is the design aspect of AnchoredDesign, present in the protocol but not showcased by this benchmark. Known-structure benchmarking of this variety is not capable of testing both backbone flexibility and design quality at the same time: there is no way to know that the natural protein is the best possible structure and sequence at the interface, and so there is no reason to believe flexible-backbone design will converge to nature's solution. Fortunately, Rosetta has also proven to be very effective at the design problem.[6], [8], [36], [50] The anchor displacement experiment suggests that in the design case, small displacements and rotations at the anchor position could be searched to increase the probability that designable interfaces are sampled.

A complete test for the AnchoredDesign protocol will be a full pass from separated target and scaffold starting structures, through computational prediction of a binding sequence, to experimental verification that the model is correct.

## Supporting Information

Figure S1 **Annotated AnchoredDesign results.** This text represents excerpts from a PDB file result from an AnchoredDesign run. Vertical or horizontal ellipses (…) indicate where text has been excised for space or section boundaries. As in most PDB files, there are many thousands of ATOM records that compose the bulk of the file (A). The next section is the Rosetta-standard score section, listing the whole-structure and per-residue scores for all scorefunction terms. After B comes the output from LoopAnalyzerMover, including per-residue listings for several statistics (along with an annotation); C demarcates skipped residue lines. The utility of LoopAnalyzerMover is in finding small loop errors; for example residue 322 has a slightly longer peptide bond (1.339 Å, pbnd_dst) than the Rosetta standard 1.329 Å. Section D includes the protein sequence (useful in design mode) followed by output from InterfaceAnalzyerMover. This includes a listing of residues with unsatisfied, buried hydrogen bonds near the interface (clipped by E) and PyMOL selections of interface residues (clipped by F). The file ends with a long list of added statistics, which also appear in the scorefile (clipped by G) (see also Table 1).
(EPS)

Figure S2 **Best scoring prediction for 8 complexes.** This figure demonstrates the relaxed crystal structure input and AnchoredDesign's lowest-score prediction for 8 of the 16 structures. The first and third rows show whole structures, and the second and fourth zoom in on the predicted loops. The nonmoving side of the interface is in green, the actual partner in cyan and the prediction in yellow. The predicted loop is red and the anchor is white. Structures are labeled with their PDB code in the lower right of each cell. For most structures, the predicted rigid-body placement and loop is indistinguishable from the relaxed crystal structure. The other 8 structures are shown similarly in Figure 5.
(TIF)

Figure S3 **LRMSD versus score plots.** This figure shows score versus LRMSD plots for each of the 16 structures. RMSD was calculated between the relaxed crystal structure (input) and AnchoredDesign's output. Plots are labeled with their PDB ID in the lower right of each cell. Black points are predictions, red points are relaxed native trajectories, and blue points are conservative relaxed native trajectories which skipped the centroid phase (see main text). Blue points may lie under red points, and red points may lie under black points. Some high-score points are out of view on all plots; all low-score points are present. On the RMSD (X) axis, the first, second, and third tic marks represent 1, 2.5, and 5 Ångstroms.
(TIF)

Figure S4 **Loop RMSD versus score plots.** This figure shows score versus loop RMSD plots for each of the 16 structures. RMSD was calculated between the relaxed crystal structure (input) and AnchoredDesign's output. Plots are labeled with their PDB ID in the lower right of each cell. Black points are predictions, red points are relaxed native trajectories, and blue points are conservative relaxed native trajectories which skipped the centroid phase (see main text). Blue points may lie under red points, and red points may lie under black points. Some high-score points are out of view on all plots; all low-score points are present. On the RMSD (X) axis, the first, second, and third tic marks represent 1, 2.5, and 5 Ångstroms. Some plots are zoomed in beyond 5 or 2.5 Ångstroms and fewer tics appear.
(TIF)

Figure S5 **2obg and 1fc4 sampling.** This image shows the range of sampling in 100 convenience-sample result structures from the standard protocol. Panel A contains 100 predictions for PDB 2obg, and panel B contains the correct 2obg structure for comparison. Panels C and D contain the same for the 1fc4 system. In each panel, the fixed side of the interface is at the bottom in green and the spread of possible docking orientations are across the top in a rainbow of colors. It can be seen that many possible interfaces and rigid body degrees of freedom are sampled. Furthermore, this sample represents result structures; many more orientations are sampled within a trajectory but rejected by Monte Carlo or superseded by better structures.
(TIF)

Table S1 **AnchoredDesign scorefunction.** This table lists the Rosetta scorefunction terms used in the benchmarking experiments for AnchoredDesign. All terms and weights except chainbreak are the standard Score12 terms used for many Rosetta experiments. Chainbreak is used with CCD loop modeling; the weight of 2 was determined empirically and can be modified by an AnchoredDesign command line flag.
(DOC)

Table S2 **Effects of anchor displacement.** This table lists the RMSD metrics (see results) for both the standard and anchor-displaced AnchoredDesign benchmarking experiment. The “standard” columns duplicate Table 3 for ease of reading; the “displaced” columns list the values from this new experiment. To produce displacement, the alpha carbon of the anchor residue (and accordingly, all other residues in the moving side of the interface) was translated to a random position within one Ångstrom in x, y, and z (an 8 square-Ångstrom cube) of its original position. To relax possible clashes caused by this displacement, the anchor was allowed to move instead of being held in its original position. It was gently constrained to its correct position using constraints were automatically generated between the anchor position's alpha carbon and the closest four alpha carbons across the interface. Each constraint was scored by a harmonic potential weighted to produce a score penalty of half a unit at 1 Ångstrom deviation. For comparison, total protein scores for these systems are in the range of hundreds to thousands, so half a score unit is weak.
(DOC)
