# Supplementary figures and images for: Anchored Design of Protein-Protein Interfaces
Source: PLoS One. 2011 Jun 17;6(6):e20872. doi: 10.1371/journal.pone.0020872 (PMC3117852; doi:10.1371/journal.pone.0020872)

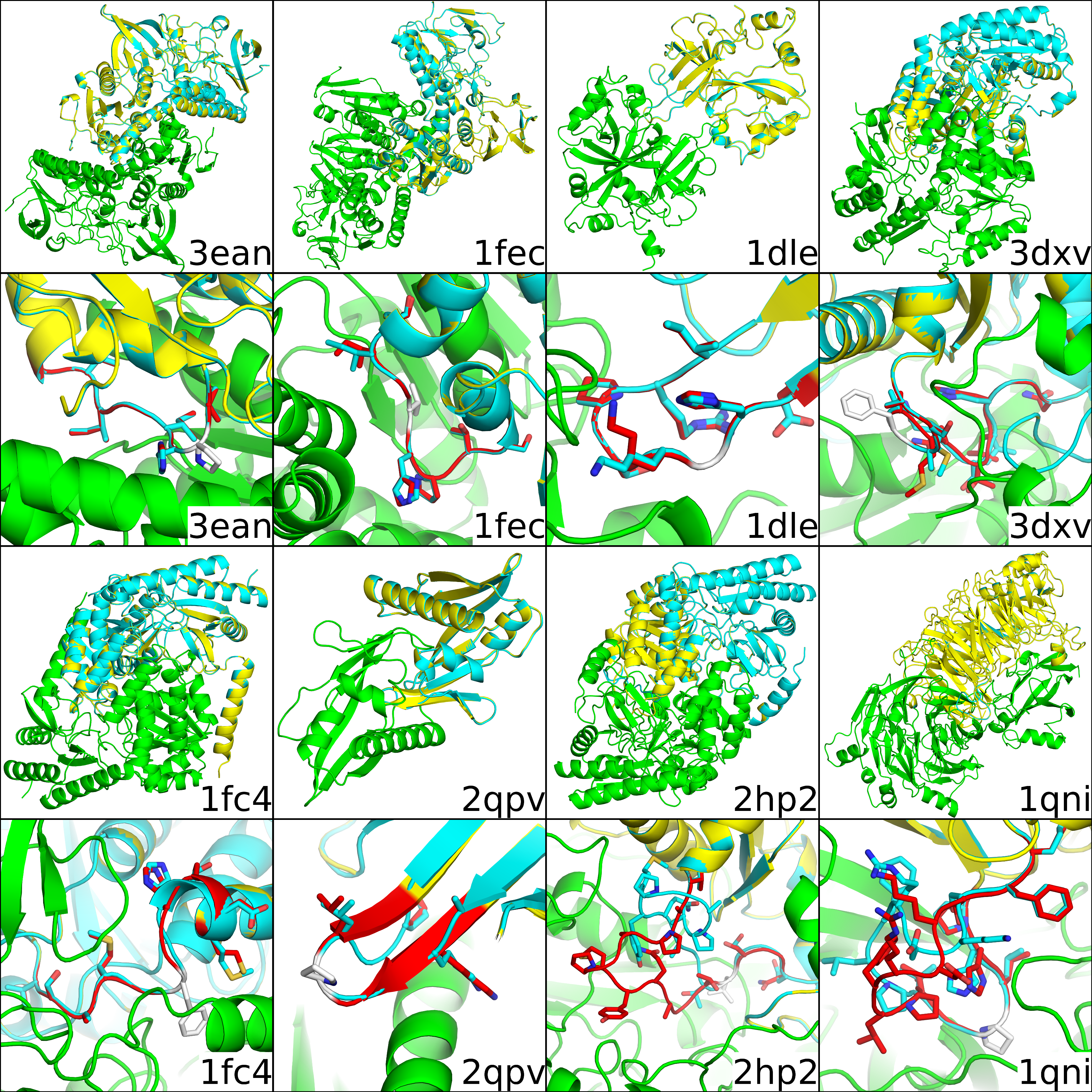

Supplement: Figure S2 — Best scoring prediction for 8 complexes. This figure demonstrates the relaxed crystal structure input and AnchoredDesign's lowest-score prediction for 8 of the 16 structures. The first and third rows show whole structures, and the second and fourth zoom in on the predicted loops. The nonmoving side of the interface is in green, the actual partner in cyan and the prediction in yellow. The predicted loop is red and the anchor is white. Structures are labeled with their PDB code in the lower right of each cell. For most structures, the predicted rigid-body placement and loop is indistinguishable from the relaxed crystal structure. The other 8 structures are shown similarly in Figure 5. (TIF) [file pone.0020872.s002.tif]

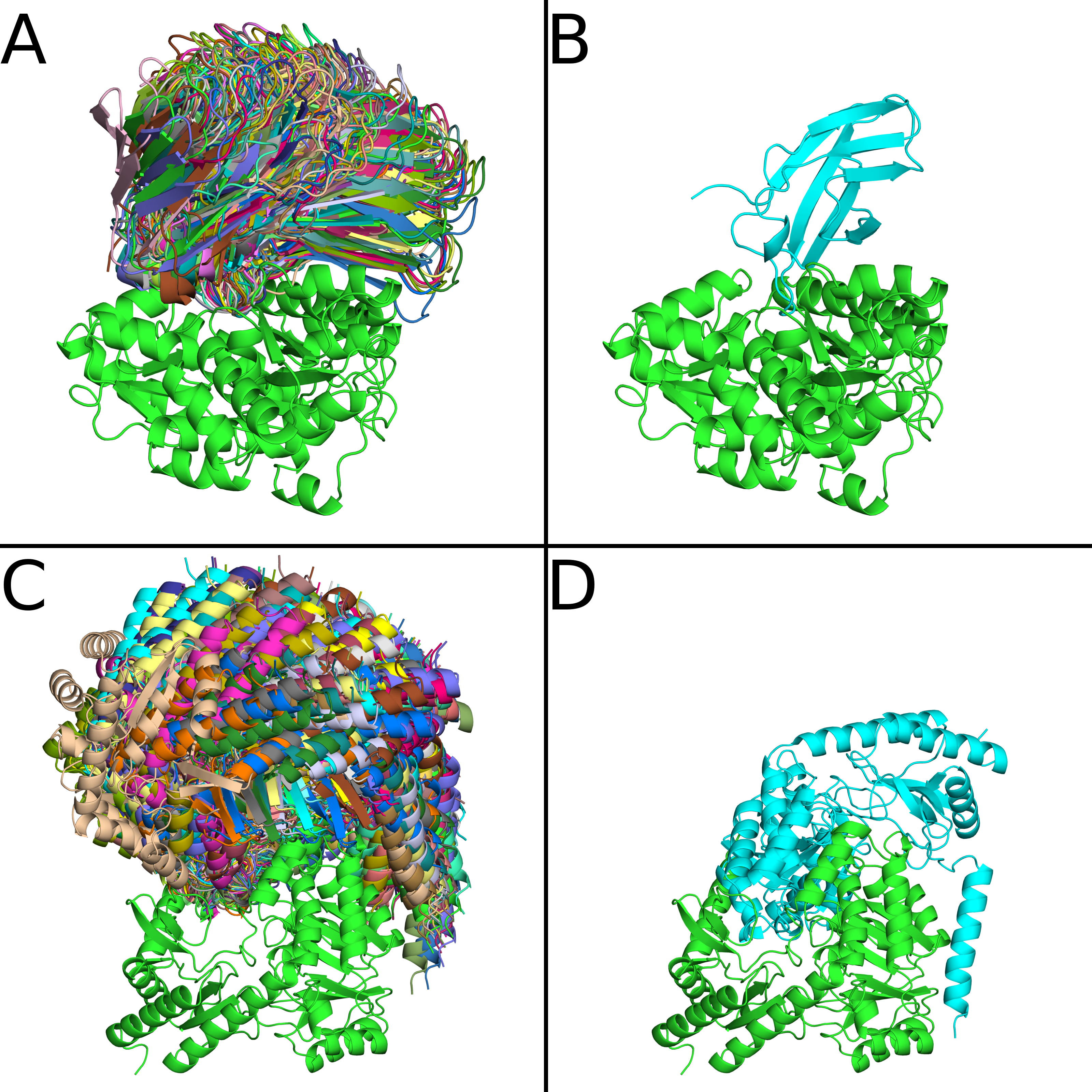

Supplement: Figure S5 — 2obg and 1fc4 sampling. This image shows the range of sampling in 100 convenience-sample result structures from the standard protocol. Panel A contains 100 predictions for PDB 2obg, and panel B contains the correct 2obg structure for comparison. Panels C and D contain the same for the 1fc4 system. In each panel, the fixed side of the interface is at the bottom in green and the spread of possible docking orientations are across the top in a rainbow of colors. It can be seen that many possible interfaces and rigid body degrees of freedom are sampled. Furthermore, this sample represents result structures; many more orientations are sampled within a trajectory but rejected by Monte Carlo or superseded by better structures. (TIF) [file pone.0020872.s005.tif]
