# Supplementary material for: Anchored Design of Protein-Protein Interfaces
Source: PLoS One. 2011 Jun 17;6(6):e20872. doi: 10.1371/journal.pone.0020872 (PMC3117852; doi:10.1371/journal.pone.0020872)
Supplement: Table S1 — AnchoredDesign scorefunction. This table lists the Rosetta scorefunction terms used in the benchmarking experiments for AnchoredDesign. All terms and weights except chainbreak are the standard Score12 terms used for many Rosetta experiments. Chainbreak is used with CCD loop modeling; the weight of 2 was determined empirically and can be modified by an AnchoredDesign command line flag. (DOC) [file pone.0020872.s006.doc]

| Scorefunction term | Weight |
| --- | --- |
| fa_atr | 0.8 |
| fa_rep | 0.44 |
| fa_sol | 0.65 |
| fa_intra_rep | 0.004 |
| pro_close | 1 |
| fa_pair | 0.49 |
| hbond_sr_bb | 0.585 |
| hbond_lr_bb | 1.17 |
| hbond_bb_sc | 1.17 |
| hbond_sc | 1.1 |
| dslf_ss_dst | 0.5 |
| dslf_cs_ang | 2 |
| dslf_ss_dih | 5 |
| dslf_ca_dih | 5 |
| rama | 0.2 |
| omega | 0.5 |
| fa_dun | 0.56 |
| p_aa_pp | 0.32 |
| ref | 1 |
| chainbreak | 2 |
