# Supplementary material for: Anchored Design of Protein-Protein Interfaces
Source: PLoS One. 2011 Jun 17;6(6):e20872. doi: 10.1371/journal.pone.0020872 (PMC3117852; doi:10.1371/journal.pone.0020872)
Supplement: Table S2 — Effects of anchor displacement. This table lists the RMSD metrics (see results) for both the standard and anchor-displaced AnchoredDesign benchmarking experiment. The “standard” columns duplicate Table 3 for ease of reading; the “displaced” columns list the values from this new experiment. To produce displacement, the alpha carbon of the anchor residue (and accordingly, all other residues in the moving side of the interface) was translated to a random position within one Ångstrom in x, y, and z (an 8 square-Ångstrom cube) of its original position. To relax possible clashes caused by this displacement, the anchor was allowed to move instead of being held in its original position. It was gently constrained to its correct position using constraints were automatically generated between the anchor position's alpha carbon and the closest four alpha carbons across the interface. Each constraint was scored by a harmonic potential weighted to produce a score penalty of half a unit at 1 Ångstrom deviation. For comparison, total protein scores for these systems are in the range of hundreds to thousands, so half a score unit is weak. (DOC) [file pone.0020872.s007.doc]

| PDB | Input loop RMSD (Å) | Input loop RMSD (Å) | Input IRMSD (Å) | Input IRMSD (Å) | Input LRMSD (Å) | Input LRMSD (Å) |
| --- | --- | --- | --- | --- | --- | --- |
|  | standard | displaced | standard | displaced | standard | displaced |
| 1dle | 0.09 | 0.18 | 0.04 | 0.07 | 0.16 | 0.16 |
| 1qni | 1.99 | 2.40 | 0.53 | 0.69 | 0.47 | 0.61 |
| 1fc4 | 0.40 | 0.40 | 0.08 | 0.09 | 0.08 | 0.11 |
| 2qpv | 0.63 | 1.13 | 0.19 | 0.39 | 0.23 | 0.52 |
| 2wya | 0.24 | 0.38 | 0.06 | 0.10 | 0.06 | 0.09 |
| 3dxv | 0.62 | 1.55 | 0.14 | 0.35 | 0.13 | 0.32 |
| 1u6e | 0.13 | 0.62 | 0.06 | 0.12 | 0.12 | 0.14 |
| 1jtp | 0.08 | 0.20 | 0.07 | 0.11 | 0.30 | 0.24 |
| 2hp2 | 3.43 | 3.29 | 1.37 | 1.06 | 1.24 | 0.97 |
| 2obg | 0.34 | 1.04 | 0.26 | 2.69 | 0.58 | 9.10 |
| 3cgc | 2.23 | 4.54 | 4.74 | 6.71 | 14.16 | 17.06 |
| 2i25 | 0.11 | 0.23 | 0.28 | 0.32 | 2.18 | 2.49 |
| 3ean | 0.06 | 0.28 | 0.03 | 0.05 | 0.06 | 0.06 |
| 1fec | 0.14 | 0.22 | 0.03 | 0.04 | 0.04 | 0.05 |
| 1zr0 | 0.17 | 0.08 | 0.14 | 0.04 | 0.47 | 0.15 |
| 2bwn | 1.30 | 4.50 | 0.30 | 4.99 | 0.29 | 11.52 |
